# Apoplexy

**Published:** 1881-09

**Authors:** 


### APOPLEXY.

[From Dr. W. W. Hall's Family Doctor.]

IS a sudden prostration of sense, motion and consciousness, in consequence of an excessive amount of blood being sent to the brain through the arteries, shown by flushed face and eyes, violent beating of the arteries, and swollen veins at the sides of the neck. The pulse is full and slow; the face is turgid, or of a dusky hue; the breathing is measured, and often of a snoring character.

There are two kinds of apoplexy: one called "congestive," where the blood vessels are very full; the blood is, as it were, impeded in them, but still remains within them. In "hemorrhagic apoplexy," the blood is forced along the arteries to such an extent that in some weak spot the vessel gives way and the blood presses out into the brain in a clot. Sometimes violent coughing or vomiting produces the same effect, the uniform result being death, but not always or necessarily; for the clot is sometimes absorbed, and on examination of the brain years after, when the person has died of some totally different disease, the proof of a previous clot is obvious.

Apoplexy seldom occurs in young persons; is much more rare in women than in men; there is an increasing liability to it with increasing years; after thirty, more and more begin to die with it, until at fifty it becomes alarmingly frequent in proportion as communities are more desperate in their effort for wealth and position and distinction, as in large cities, especially in New York, where the struggle for money sweeps all other considerations aside, and everything is made to yield to its accumulation. A man's thoughts are active and keen in proportion to the supply of good arterial blood to the brain; at the same time deep thought attracts blood there in unusual quantities, and stimulants of all kinds increase the flow of the blood to the brain, hence they are said to "excite" us. Heavy feeding does the same thing by causing more blood to be made, hence the blood-vessels of the brain must convey more, and require a greater force and capacity and strength to carry the increased quantity. Hence gluttonous persons and wine-bibbers, gourmands and liquor-drinkers are peculiarly liable to apoplexy; so are public speakers, because the brain is all alive to the subject, and this very activity invites the blood there in increasing quantities, and everything which does that invites apoplexy, such as a heavy strain—all see how the blood gathers in the face under such a strain—or in running or wrestling, or in any of the active games; violent bursts of anger do the same thing, or great emotions, of whatever sort. Hence, the wisdom of all—especially after fifty—is in seeking repose, avoiding all strains and liftings and rapid motions, all laborious efforts, physical or mental; avoid all running up stairs, all running to catch a public vehicle; cultivate also a quiet mind, a calmness and sobriety of deportment. By so doing, and maintaining "the general health," persons liable to apoplexy, or of an apoplectic make and age, stoutness of build, shortness of neck, with large features, may indefinitely postpone an attack of apoplexy, and finally die of some very different disease. Any man over fifty, whether he be short and thick, or tall and slim, is liable to an attack of apoplexy any day, if in his previous life he has studied hard and long, or studied severely for a short period, or has been engaged in great enterprises, or in weighty and responsible avocations, bringing a strong, steady and protracted strain on the brain.

As apoplexy is almost always fatal, it will save a great deal of unnecessary trouble and apprehension to be able to decide at once, when a person suddenly becomes unconscious, whether it is apoplexy or not.

First. If it be preceded by an altered condition of the kidneys or bladder, whereby the proper amount of urine has not been passed from the system, and has been reabsorbed to poison the blood and cause diseased conditions in various parts of the body, then the disease is not real apoplexy, but is uræmia, which means that the urine is mixed with the blood and caused the attack in question; if, under such circumstances, attention is paid to the urinary organs the patient may soon get well and permanently so.

Second. If the person is "dead drunk," the ailment is of alcoholic origin, known by the smell of the breath and other attendant circumstances, and hence is not really apoplectic.

Third. There may be this "coma" or insensibility from narcotic poison, accidental or deliberate, which has been induced by taking laudanum, morphia or kindred articles; if that is the case, the pupil of the eye is contracted; if the attack has resulted from some other poisoning the pupils, on the contrary, are dilated; in such cases, by using "antidotes" the person may recover in a short time, and by next day be as well as ever.

Fourth. Insensibility, coma, may result from a stroke on the head or a fall, which is to be determined by a history of the case, or by appearances of things around the patient.

Fifth. It may be a common fainting fit, but in that case the lips are blue, the surface of the skin cold, there is no pulse, the breathing is interfered with, or there seems to be no breathing at all.

Sixth. If it is a sunstroke the pulse is feeble, skin dry and hot.

Seventh. If it is catelepsy the muscles are rigid, everything remains as at the instant of attack, the pulse is rapid, the attack soon passes off and returns again repeatedly, without any paralysis of any part. In short, apoplexy has a slow pulse, noisy breathing, and stupor. Epilepsy has a quick pulse and convulsive motion of the limbs. Fainting has no pulse, no perceptible breathing and a pale face.

### TREATMENT.

In an attack of real apoplexy there is not much to be done. The first thing is to send for a physician ; the next is to do what is possible to give temporary relief, but go about it with a certain degree of hopefulness and confidence, because if the person is thirty, or not much over, whatever may be the form of the apoplexy, he may get well, especially if the constitution be not broken.

The first thing to be done is to have the patient sit up, so as to allow the blood to fall down from the head by its own gravity ; in addition, use more active means to draw the blood from the head, by putting large mustard plasters to the legs and back and belly, one after another ; use a speedy injection during the attack, so as to unload the bowels, and as soon as possible afterwards give a dose of salts or castor oil so as to clean out the bowels. If there be much hair, cut or shave it off at once, and place a bag of pounded ice over the scalp, or keep cloths dipped in cold water, pressed out slightly so that the water shall not dribble on the clothing, applied to the scalp ; renew these cloths every five minutes until the head seems to be of the natural warmth. In all cases administer an active purgative, one tablespoon of castor oil every hour, or a tablespoon of salts every hour, until the bowels act freely.

But as apopletic attacks are becoming so frequent in comparison with past years, and in consequence of a generally prevalent straining of the brain in conducting the affairs of life, every professional man after fifty, every man engaged in responsible employments, every student, every literary man, all sedentary persons, should consider themselves liable to apoplexy, either by acquired or hereditary conditions, and use these preventive measures habitually, systematically, and persistently, which have been already stated, giving special prominence to :

First. Light and early suppers.

Second. One free, full evacuation of the bowels every twenty-four hours.

Third. Avoid the use of intoxicating drinks late at night ; *or at any time.*

Fourth. Avoid all sudden and violent outbursts of passion.

Fifth. Guard against hasty, violent, or long protracted exercise.

